# Hereditary Cancer Gene Panel Testing in a Croatian Cohort: The First Results from the University Hospital of Split

**DOI:** 10.3390/medicina62101870

**Published:** 2026-09-27

**Authors:** Tomislav Smoljo, Marin Ogorevc, Toni Čeprnja, Danijel Antonio Grubišić, Dora Knezović, Ante Tavra, Nenad Kunac, Bernarda Lozić, Eduard Vrdoljak

**Affiliations:** 1 Department of Oncology, University Hospital of Split, 21000 Split, Croatia; 2 Department of Pathology, Forensic Medicine, and Cytology, University Hospital of Split, 21000 Split, Croatia; mogorevc@kbsplit.hr (M.O.);; 3 Section of Medical Genetics, Department of Pediatrics, University Hospital of Split, 21000 Split, Croatia; 4 School of Medicine, University of Split, 21000 Split, Croatia

**Keywords:** hereditary cancer syndromes, genetic predisposition to disease, high-throughput nucleotide sequencing, germ-line mutation, panel testing

## Abstract

*Background and Objectives*: Hereditary cancer predisposition is associated with pathogenic germline variants in numerous genes. Identification of inherited cancer susceptibility enables personalized screening, preventive interventions, and targeted therapies. It also facilitates cascade testing of relatives who may be at increased risk and benefit from tailored clinical management. We describe the first results of hereditary cancer patient identification, selection, and genetic testing at the University Hospital of Split. *Materials and Methods*: Next-generation sequencing and hereditary cancer gene panel analysis were performed in individuals who met the National Comprehensive Cancer Network clinical criteria for genetic testing at the University Hospital of Split between May 2025 and July 2026. A comprehensive cancer gene panel covering 101 genes was used. *Results*: A total of 297 individuals underwent genetic testing, including 268 (90.24%) females and 29 (9.76%) males. Of these, 138 (46.46%) were unaffected individuals who met testing criteria based on a family history of cancer. Among individuals with a personal history of malignancy, breast cancer was the most common diagnosis, occurring in 130 patients (43.77%). Pathogenic or likely pathogenic (P/LP) variants, as well as risk variants, were identified in 80 individuals (26.94%), including seven individuals carrying two P/LP variants. Variants were detected in 29 different genes, most frequently *BRCA1* (*n* = 14), *BRCA2* (*n* = 11), *CHEK2* (*n* = 9) and *PALB2* (*n* = 7). The detected variant types included frameshift (*n* = 30), missense (*n* = 22), nonsense (*n* = 21), splice-site variants (*n* = 4), start loss variants (*n* = 4), large deletions (*n* = 4), and in-frame deletions (*n* = 2). *Conclusions*: To our knowledge, this is the first study to report the results of panel germline testing for hereditary cancer syndromes in a Croatian population. These findings provide initial epidemiological data on the spectrum and distribution of P/LP variants. The relatively high detection rate may reflect the recent implementation of genetic testing and the careful selection of individuals with a strong clinical suspicion of hereditary cancer predisposition.

## 1. Introduction

Hereditary cancer syndromes are characterized by an inherited predisposition to malignancy caused by pathogenic germline variants in specific genes, conferring a higher lifetime cancer risk compared with the general population. It is estimated that approximately 5–10% of all cancers arise in individuals with such genetic susceptibility [1]. In patients with a personal history of cancer, a hereditary cancer syndrome is suspected when cancer is diagnosed at an early age, when a patient is affected by multiple primary cancers, or when multiple family members are affected. Clinical guidelines, such as the National Comprehensive Cancer Network (NCCN) guidelines, have been established to identify individuals at risk who should be offered genetic testing [2]. Identification of pathogenic variants underlying tumor development enables personalized approaches to treatment, surveillance, risk-reducing interventions, and genetic counseling [3].

Beyond affected individuals, genetic testing facilitates the identification of at-risk relatives who may benefit from enhanced surveillance and preventive strategies despite lacking a personal history of cancer. Early recognition of hereditary predisposition supports the implementation of personalized screening protocols and risk-reduction measures, with the potential to decrease cancer incidence and improve clinical outcomes [4]. Cancer risk-reducing strategies may also be applied at the population level through population-based cohort studies, thereby helping to optimize national cancer management strategies [1,5].

Advances in genomic technologies, particularly the widespread adoption of next-generation sequencing (NGS), have transformed hereditary cancer testing. NGS enables high-throughput, parallel analysis of multiple genes or genomic regions, substantially increasing diagnostic yield compared with single-gene approaches. Inclusion of additional clinically actionable genes improves identification of at-risk patients because of phenotypic overlap among hereditary cancer syndromes [6,7]. Compared with targeted *BRCA1* and *BRCA2* testing, multigene panel testing in hereditary breast and ovarian cancer cohorts enables comprehensive detection of clinically relevant variants in additional genes, thereby refining risk assessment and informing precision oncology strategies [8].

In this study, we analyzed results collected during the first 15 months of hereditary cancer gene panel testing at the University Hospital of Split. In a cohort of individuals who met clinical criteria for testing, we describe demographic characteristics, personal cancer histories, the molecular pathology of detected pathogenic variants, and specific genetic features of the population from southern Croatia.

## 2. Materials and Methods

### 2.1. Study Population

The population of this retrospective study consisted of individuals with a cancer diagnosis and asymptomatic individuals who were selected based on NCCN guidelines (available at https://www.nccn.org/guidelines/category_2 accessed on 24 September 2026) and referred to the Division of Molecular Pathology of the Department of Pathology at the University Hospital of Split. After obtaining the basic personal and family history regarding malignancies needed for the proper classification of variants, a peripheral blood sample was collected for genetic analysis.

### 2.2. Sequencing and Variant Interpretation

Genomic DNA was extracted from the aforementioned samples using the QuickGene-AutoS DNA Blood Kit (Kurabo, Osaka, Japan). DNA concentration was assessed using the Qubit 4 Fluorometer and the Qubit dsDNA Assay Kit (Invitrogen, Carlsbad, CA, USA), while DNA quality was assessed using the NanoDrop SP-500 (Cole-Parmer, Vernon Hills, IL, USA). Sequencing libraries were prepared using the Magnis SureSelect XT HS Kit (Agilent Technologies, Santa Clara, CA, USA) on the Magnis NGS Prep System (Agilent Technologies, Santa Clara, CA, USA). The kit utilizes increased probe densities for difficult-to-enrich regions and custom target enrichment probes to ensure comprehensive coverage of both coding and non-coding regions. Library quality and fragment size distribution were evaluated with the TapeStation 4150 System (Agilent Technologies, Santa Clara, CA, USA). Paired-end 150 bp sequencing was performed on the DNBSEQ-G400 sequencer (MGI Tech Co., Shenzhen, China), with a minimum coverage of 100×. The obtained raw data were analyzed using the SeqOne platform (SeqOne, Montpellier, France). Analysis in all patients included 101 genes (Supplementary Table S1) covered by the customized comprehensive cancer gene panel. Sequencing reads were aligned to the human reference genome (GRCh37) using the BWA-MEM2 algorithm (v2.2.1) [9]. Germline single-nucleotide variants (SNVs) and small insertions/deletions (indels) were identified using the Genome Analysis Toolkit (GATK) version 4.6.0.0 [10]. Germline copy number variants (CNVs) were detected using the CNVCapture algorithm version 2.5.4 (SeqOne, Montpellier, France) and analysis of the coverage plots (Supplementary Figure S1). For CNV calling, control samples were selected automatically from the other samples in the same sequencing run and previous runs (up to 96 samples total), using a correlation of coverage profiles of ≥0.9 as the inclusion criterion, with a minimum of six control samples required. Samples for which fewer than six suitable controls were found were classified as failing CNV calling. Region-level quality was assessed from the variability of copy number ratios across samples. Regions with a coefficient of variation > 0.2 were classified as uncallable, since inter-sample variability was too high to distinguish true copy number changes from noise. When a failed sample or an uncallable region involved a gene relevant to the patient’s clinical indication, sequencing was repeated in a separate run, and CNV analysis was repeated using the new control set. The laboratory’s quality control (QC) thresholds were set as a mean coverage of at least 300×, >99% of target regions attaining at least 30× sequence coverage in post-filtering bases, and >95% of target regions attaining at least 100× sequence coverage in post-filtering bases. Targeted enrichment during library preparation and increased sequencing depth thresholds were used to address regions with inadequate coverage.

Detected variants were classified according to the ACMG guidelines for variant interpretation [11] and ACGS guidelines for variant classification [12]. Gene-specific criteria for variant interpretation given by ClinGen gene curation expert panels were applied if available [13,14,15,16,17,18,19]. Variants that were classified as pathogenic or likely pathogenic, as well as risk alleles, were included in our study as a positive result.

### 2.3. Ethics Declaration and Ethical Approval

This study protocol was approved by the Ethics Committee of the University Hospital of Split under approval number 2181-147/01-06/LJ.Z.-26-02. All personal identifiers of participants were de-identified, and all methods were performed in accordance with the Declaration of Helsinki.

## 3. Results

### 3.1. Demographic Characteristics of Study Cohort

The study cohort included 297 individuals who met clinical criteria for genetic testing at the University Hospital of Split between May 2025 and July 2026, of whom 268 (90.24%) were female and 29 (9.76%) were male. Notably, 159 (53.54%) participants were patients with a diagnosis of malignant disease, including 130 (43.7%) individuals with breast cancer as the most prevalent diagnosis in the cohort. In total, 138 (46.46%) participants met the testing criteria because of a family history of cancer, including 122 (41.08%) asymptomatic individuals and an additional 16 (5.39%) individuals with a diagnosis of a benign tumor. The median age at the time of testing was 48 years, and 3 tested individuals were younger than 18 years.

### 3.2. Detected Variants, Affected Genes, and Molecular Pathology

A heterozygous pathogenic or likely pathogenic variant, as well as risk variants, was found in 80 (26.94%) tested individuals, while 7 (2.36%) of them carried heterozygous P/LP variants in two different genes (Supplementary Table S2). Additionally, variants of uncertain significance (VUS) were detected in 24 (8.08%) individuals. In total, 72 (90%) individuals with a positive test result were female, and 8 (10%) were male. Among individuals with positive test results, 26 (32.5%) were asymptomatic, 4 (5%) were diagnosed with a benign tumor, and 51 (63.75%) had a malignant tumor, of which breast cancer was the most frequent diagnosis, with 38 (47.5%) patients. The median age at the time of testing was 49.5 years, while two positive individuals were younger than 18 years.

Variants were detected across 29 different genes. Clinically actionable variants were detected in 46 individuals and included the following genes: *BRCA1* (*n* = 14), *BRCA2* (*n* = 11), *PALB2* (*n* = 7), *CDKN2A* (*n* = 4), *TP53* (*n* = 2), *APC* (*n* = 1), *EPCAM* (*n* = 1), *LZTR1* (*n* = 1), *MLH1* (*n* = 1), *MSH6* (*n* = 1), *PMS2* (*n* = 1), *TSC1* (*n* = 1), and *TSC2* (*n* = 1). A detailed list of all the detected variants can be found in Supplementary Table S3.

Analysis of the affected genes in participants grouped according to the clinical condition showed that, among the 38 participants with breast cancer, the most frequently affected genes were *BRCA1* (*n* = 7) and *CHEK2* (*n* = 7), followed by *PALB2* (*n* = 6), *BRCA2* (*n* = 4), *NTHL1* (*n* = 3), *NBN* (*n* = 2), *MUTYH* (*n* = 2), and *TP53* (*n* = 2), while *BARD1*, *DDX41*, *ERCC3*, *FANCC*, *FANCL*, *FANCM*, *MSH6*, and *POT1* were found only in singular cases. An interesting case is that of a young adult patient with a synchronous occurrence of breast cancer and chondrosarcoma in whom a whole-gene deletion of the *TP53* gene and a pathogenic *CHEK2* variant were found. The whole-gene deletion was classified as pathogenic based on ClinGen and ACGS criteria 1A, 2A, and 5G. In the group of 26 asymptomatic participants, 7 were carriers of a pathogenic variant in the *BRCA2* gene and 6 in the *BRCA1* gene, three in *FANCL*, and two in *ATM* and *CHEK2*, while *APC*, *CDKN2A*, *FANCD2*, *HOXB13*, *MUTYH*, *PALB2*, *PMS2,* and *RAD51C* variants were found in one participant each, as shown in Figure 1.

The detected variant types are presented in Table 1.

### 3.3. Recurring Variants

Some of the detected pathogenic variants recurred in our cohort. The most frequent variants were detected in 6 participants each: the c.5266dup variant in the *BRCA1* gene was detected in 6 participants, including 3 individuals with breast cancer and 3 asymptomatic individuals, and the c.470T>C variant in the *CHEK2* gene was detected in 6 participants with breast cancer. The most frequent variant in the *BRCA2* gene was the c.6641dup variant, detected in 2 participants with breast cancer and 3 asymptomatic individuals. Individuals with the most frequent variants in our cohort are presented in Table 2.

### 3.4. Novel Variants

Detailed analysis of the detected variants revealed two variants that had not previously been reported in the ClinVar, PubMed, or Leiden Open Variation databases at the time of analysis (16 August 2026). Table 3 summarizes information on the novel variants, including the affected gene, variant type, variant classification, classification criteria applied, diagnosis, other affected genes in the variant carrier, and the age of the carrier. Segregation analysis was not performed for the *MLH1* gene variant because the parents were deceased, but family history was positive for Lynch-associated cancers, whereas for the *TSC2* gene variant, family segregation analysis was performed, which determined that the variant occurred de novo.

## 4. Discussion

In this study, we analyzed the results from the first 15 months of hereditary cancer gene panel testing at the University Hospital of Split. To our knowledge, this is the first cohort study using a hereditary cancer gene panel in the Croatian population. The study cohort consisted of 297 individuals who met clinical criteria for genetic testing between May 2025 and July 2026. Similar to the other studies, most of the participants were female, while breast cancer was the most frequent diagnosis [20,21,22]. This over-representation of female breast cancer patients may be explained by the fact that breast cancer is the most frequent cancer in females and hereditary breast and ovarian cancer syndrome is the most frequent hereditary cancer syndrome [3].

The high proportion of positive results (26.94%) compared with other similar studies [7,23,24] may be explained by the recent implementation of this testing method in our institution and the selective inclusion of individuals with a strong clinical suspicion of hereditary cancer syndromes. Additionally, analysis of individuals with positive test results revealed that seven participants carried two P/LP variants. Compared with the overall cohort, individuals with positive test results had a higher proportion of cancer diagnoses, and only 32.5% were asymptomatic.

Similar to previous reports [7], frameshift mutations were the most prevalent variant type, followed by missense and nonsense variants, while splicing variants, start loss variants, large deletions, and in-frame deletions were detected less frequently, as presented in Table 1.

Previous studies have reported that multi-gene panels increased the detection rate compared with analysis limited to a small number of genes [6,7,8,25,26]. In line with these findings, pathogenic variants in this study were detected in 29 of the 101 genes included in the panel, comprising 25 variants in *BRCA1* and *BRCA2* and 62 variants in other genes.

Although our sample size is small, it is noteworthy that the *CHEK2* and *PALB2* genes were more frequently affected in the breast cancer population (*n* = 7 and 6, respectively). In asymptomatic participants, there was a high proportion of carriers of pathogenic variants in *BRCA1* and *BRCA2* (*n* = 6 and 7, respectively), as presented in Figure 1.

As this study is, to our knowledge, the first hereditary cancer gene panel cohort study conducted in the Croatian population, analysis of the most frequent pathogenic variants is of particular interest. The most frequent variants in our cohort are presented in Table 2. The most frequent variant, *BRCA1*:c.5266dup, identified in three breast cancer patients and three unaffected individuals in our cohort, has previously been reported in the Croatian population [27,28]. This variant was originally described as a founder mutation in the Ashkenazi Jewish population [29] but later shown to originate from a common ancestor in northern Europe approximately 1800 years ago [30].

The most frequent variant in the *BRCA2* gene in our cohort, c.6641 dup, identified in two breast cancer patients and three unaffected individuals, was previously described in the Croatian population [28]. The second most common *BRCA2* variant in our cohort, c.9371A>T, identified in one breast cancer patient and in two unaffected individuals, has also been reported in the Croatian population [28].

The *CHEK2* c.470T>C variant, known as a low-penetrance variant, was detected in six breast cancer participants in our cohort and has previously been reported as a Croatian founder risk variant for testicular germ cell tumors [31,32]. ACMG/AMP criteria BS1 and PS3_Mod were used for this variant classification as per the CanVIG-UK *CHEK2* Gene-Specific Guidance (Version 1.3). While the criteria themselves would classify the variant as a VUS, a meta-analysis has found a significant association between carrying the variant and an increased risk of breast cancer (OR = 1.48, 95% CI = 1.31–1.66, *p* < 0.0001); therefore, we classified it as a risk allele [33].

The identification of novel variants is important for determining specific phenotypic features of the diagnosed disease, especially in the population from which our cohort originated. Novel variants in our cohort include a large deletion and a frameshift variant. A large deletion in the *MLH1* gene was detected in a young adult patient with colorectal cancer and a positive family history. Pathogenic variants in the *MLH1* gene cause Lynch syndrome 2. A frameshift variant in the *TSC2* gene was identified in a newborn with clinical suspicion of tuberous sclerosis and a negative family history of the disease. Tuberous sclerosis 2 is inherited in an autosomal dominant manner, but only one third of individuals with tuberous sclerosis have an affected parent, whereas two-thirds carry a de novo pathogenic variant, with a higher proportion of de novo variants occurring in the *TSC2* gene compared with the *TSC1* gene [34].

A rarely described whole-gene deletion of the *TP53* gene was found in a young adult patient with synchronous occurrence of breast cancer and chondrosarcoma. Heterozygous germline pathogenic variants in *TP53* cause Li–Fraumeni syndrome, and heterozygous whole-gene deletions were previously reported in patients with Li–Fraumeni syndrome [35,36,37,38].

One limitation of this study is that most participants were from Southern Croatia, as testing was performed at a single center, the University Hospital of Split. Possible intronic variants may have remained undetected because the testing method included only exons and exon–intron junctions. In addition, while repeat sequencing addressed regions and samples that failed QC, SNV and CNV calls that passed QC were not confirmed by an orthogonal method, such as Sanger sequencing or multiplex ligation-dependent probe amplification. The analytical sensitivity and specificity of variant detection methods in our cohort were therefore not independently established, and false-negative and false-positive calls cannot be excluded, particularly for small exon-level events. Cascade testing was not performed at the time of testing in most individuals; therefore, it was not included in the analysis. Ancestry was defined by nationality recorded in the hospital registry, and genetic ancestry was not assessed; therefore, some ancestral heterogeneity within the cohort cannot be excluded.

## 5. Conclusions

In conclusion, our study presents the results of the implementation of hereditary cancer gene panel testing and provides the first reported findings obtained using this testing method in the Croatian population. Carrier follow-up, assessment of cancer outcomes, testing of relatives, and VUS reclassification would provide additional valuable information regarding the clinical implications of pathogenic variant carriership in our population. These aspects represent important areas for future research.

## Figures and Tables

**Figure 1:**
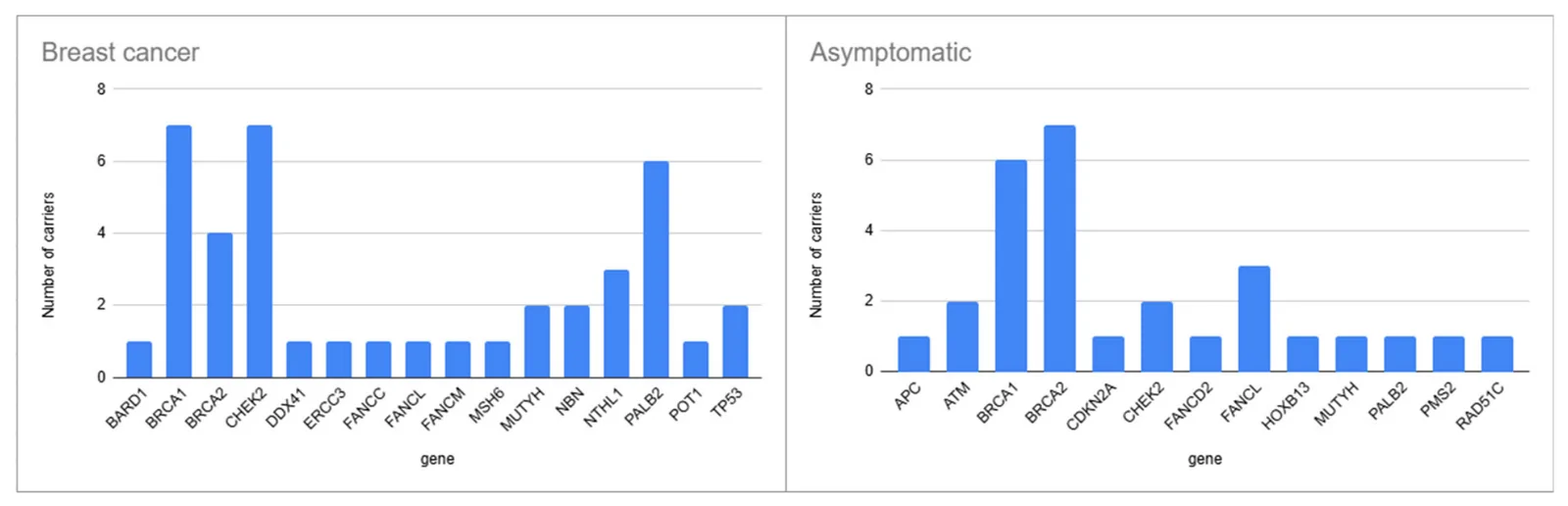
Distribution of affected genes in individuals with breast cancer compared with asymptomatic individuals.

**Table 1:** Variant types of detected pathogenic and likely pathogenic variants.

Variant Type	Number	Proportion
frameshift	30	34.48%
missense	22	25.29%
nonsense	21	24.14%
splicing	4	4.60%
start loss	4	4.60%
large deletion	4	4.60%
in-frame deletion	2	2.30%
total	87	

**Table 2:** Variants detected in three or more individuals.

Gene	Variant	Number of Carriers	Carriers with Cancer
*BRCA1*	c.5266dup	6	3 breast cancer
*BRCA2*	c.6641dup	5	2 breast cancer
*CHEK2*	c.470T>C	6	6 breast cancer
*NTHL1*	c.244C>T	4	3 breast cancer
*BRCA2*	c.9371A>T	3	1 breast cancer
*CDKN2A*	c.250G>A	3	1 colorectal cancer 1 melanoma
*PALB2*	c.395del	3	3 breast cancer
*FANCL*	c.2T>C	3	1 breast cancer

**Table 3:** Novel variants in our cohort.

Gene	Variant	Variant Type	Variant Classification	Classification Criteria Applied	Patient’s Diagnosis	Age (Years)
*MLH1*	NM_000249.4: c.(677 + 1_678-1) (1667 + 1_1668-1)del, p.?	large deletion	pathogenic	CanVIG-UK MMR Gene-Specific Guidance: PVS1, PM2_Sup, PP4_Sup	Colorectal cancer	29
*TSC2*	NM_000548.5: c.3733del, p.(Arg1245GlyfsTer80)	frameshift	pathogenic	CanVIG-UK Consensus Specifications: PVS1, PM2, PM5_Sup	Tuberous sclerosis	0

## Data Availability

The raw data supporting the conclusions of this article will be made available by the authors upon request.

## Supplementary Materials

The following supporting information can be downloaded at https://www.mdpi.com/article/10.3390/medicina62101870/s1, Table S1: The list of genes included in the gene panel; Table S2: Carriers of 2 variants reported as a positive finding; Table S3: List of all variants reported as a positive finding in our cohort; Figure S1: Coverage plots for the detected copy number variants.

## Abbreviations

The following abbreviations are used in this manuscript:

CNV	Copy-number variant
NGS	Next-generation sequencing
LP	Likely pathogenic
P	Pathogenic
NCCN	National Comprehensive Cancer Network
VUS	Variant of uncertain significance
QC	Quality control
