# Supplementary material for: Hereditary Cancer Gene Panel Testing in a Croatian Cohort: The First Results from the University Hospital of Split
Source: Medicina (Kaunas). 2026 Sep 27;62(10):1870. doi: 10.3390/medicina62101870 (PMC13707095; doi:10.3390/medicina62101870)
Supplement: Supplementary file 1 [file medicina-62-01870-s001.zip › Supplementary Table S1.pdf]

Supplementary Table S1. The List of Genes Included in the Gene Panel

Gene	OMIM number	Disease
AKT1	* 164730	Cowden syndrome 6 (AD)
APC	* 611731	Adenomatous polyposis coli (AD), Brain tumor-polyposis syndrome 2 (AD), Desmoid disease, hereditary (AD), Gardner syndrome (AD), Gastric adenocarcinoma and proximal polyposis of the stomach (AD)
ATM	* 607585	Breast cancer susceptibility (AD), Ataxia-telangiectasia (AR)
AXIN2	* 604025	Oligodontia-colorectal cancer syndrome (AD)
BAP1	* 603089	Uveal melanoma susceptibility 2 (AD), Kury-Isidor syndrome (AD), Tumor predisposition syndrome 1 (AD)
BARD1	* 601593	Breast cancer susceptibility (AD)
BLM (*RECQL3*)	* 604610	Bloom syndrome (AR)
BMPR1A	* 601299	Polyposis syndrome, hereditary mixed, 2, Polyposis, juvenile intestinal (AD)
BRCA1	* 113705	Breast-ovarian cancer, familial 1, (AD), Pancreatic cancer susceptibility 4, Fanconi anemia, complementation group S (AR)
BRCA2	* 600185	Breast cancer, male susceptibility (AD), Breast-ovarian cancer, familial 2 (AD), Glioblastoma 3 (AR), Medulloblastoma (AD, AR), Pancreatic cancer 2, Prostate cancer (AD), Fanconi anemia complementation group D (AR), Wilms tumor (AD)
BRIP1	* 605882	Breast cancer, early-onset, susceptibility (AD), Fanconi anemia, complementation group J
CASR	* 601199	Epilepsy idiopathic generalized, susceptibility 8 (AD), Hyperparathyroidism, neonatal (AD, AR), Hypocalcemia (AD) Hypocalcemia with Bartter syndrome (AD), Hypocalciuric hypercalcemia, type I (AD)
CDC73	* 607393	Hyperparathyroidism-jaw tumor syndrome (AD), Hyperparathyroidism, familial primary (AD), Parathyroid adenoma with cystic changes (AD), Parathyroid carcinoma
CDH1	* 192090	Blepharocheilodontic syndrome 1 (AD), Diffuse gastric and lobular breast cancer syndrome with or without cleft lip and/or palate (AD)
CDK4	* 123829	Melanoma 3 (AD), Microcephaly 31, primary (AR)
CDKN1B	* 600778	Multiple endocrine neoplasia, type IV (AD)
CDKN2A	* 600160	Melanoma and neural system tumor syndrome (AD), Melanoma-pancreatic cancer syndrome (AD), Melanoma 2 (AD)

CEBPA	* 116897	Leukemia, acute myeloid (AD)
CHEK2	* 604373	Tumor predisposition syndrome 4, breast/prostate/colorectal
CTNNA1	* 116805	Macular dystrophy patterned 2 (AD)
CTRC	* 601405	Pancreatitis chronic susceptibility (AD)
DDX41	* 608170	{Myeloproliferative/lymphoproliferative neoplasms (AD)
DICER1	* 606241	Goiter, multinodular 1, with or without Sertoli-Leydig cell tumors (AD), Pleuropulmonary blastoma (AD), Rhabdomyosarcoma, embryonal 2
DIS3L2	* 614184	Perlman syndrome (AR)
EPCAM	* 185535	Diarrhea 5, with tufting enteropathy, congenital (AR), Lynch syndrome 8 (AD)
ERCC1	* 126380	Cerebrooculofacioskeletal syndrome 4 (AR)
ERCC2	* 126340	Cerebrooculofacioskeletal syndrome 2 (AR), Trichothiodystrophy 1, photosensitive (AR), Xeroderma pigmentosum, group D (AR)
ERCC3	* 133510	Trichothiodystrophy 2, photosensitive (AR), Xeroderma pigmentosum, group B (AR)
ERCC4	* 133520	Fanconi anemia, complementation group Q (AR), Xeroderma pigmentosum, group F (AR), Xeroderma pigmentosum, type F/Cockayne syndrome (AR), XFE progeroid syndrome (AR)
ERCC5	* 133530	Cerebrooculofacioskeletal syndrome 3 (AR), Xeroderma pigmentosum, group G (AR), Xeroderma pigmentosum, group G/Cockayne syndrome (AR)
FANCA	* 607139	Fanconi anemia, complementation group A (AR)
FANCC	* 613899	Fanconi anemia, complementation group C (AR)
FANCD2	* 613984	Fanconi anemia, complementation group D2 (AR)
FANCE	* 613976	Fanconi anemia, complementation group E (AR)
FANCF	* 613897	Fanconi anemia, complementation group F (AR)
FANCG	* 602956	Fanconi anemia, complementation group G (AR)
FANCI	* 611360	Fanconi anemia, complementation group I (AR)
FANCL	* 608111	Fanconi anemia, complementation group L (AR)
FANCM	* 609644	Premature ovarian failure 15 (AR), Spermatogenic failure 28 (AR)
FH	* 136850	Fumarase deficiency (AR), Leiomyomatosis and renal cell cancer (AD)
FLCN	* 607273	Birt-Hogg-Dube syndrome (AD), Pneumothorax, primary spontaneous (AD)

GATA2	* 137295	Leukemia, acute myeloid, susceptibility (AD), Myelodysplastic syndrome, susceptibility, Emberger syndrome (AD), Immunodeficiency 21 (AD)
GPC3	* 300037	Simpson-Golabi-Behmel syndrome, type 1 (XLR)
GREM1	* 603054	Hereditary mixed polyposis syndrome (AD)
HOXB13	* 604607	Prostate cancer, hereditary, 9
KIT	* 164920	Gastrointestinal stromal tumor, familial (AD), Mastocytosis, cutaneous (AD), Piebaldism (AD)
LZTR1	* 600574	Schwannomatosis-2, susceptibility (AD), Noonan syndrome 10 (AD), Noonan syndrome 2 (AR)
MAX	* 154950	Pheochromocytoma, susceptibility (AD), Polydactyly-macrocephaly syndrome (AD)
MEN1	* 613733	Multiple endocrine neoplasia 1 (AD)
MET	* 164860	Arthrogryposis, distal, type 11 (AD), Deafness (AR), Osteofibrous dysplasia susceptibility (AD)
MITF	* 156845	Melanoma susceptibility 8, COMMAD syndrome (AR), Tietz albinism-deafness syndrome (AD), Waardenburg syndrome, type 2A (AD)
MLH1	* 120436	Lynch syndrome 2, Mismatch repair cancer syndrome 1 (AR), Muir-Torre syndrome (AD)
MRE11A	* 600814	Ataxia-telangiectasia-like disorder 1 (AR)
MSH2	* 609309	Lynch syndrome 1 (AD), Mismatch repair cancer syndrome 2 (AR), Muir-Torre syndrome (AD)
MSH3	* 600887	Familial adenomatous polyposis 4 (AR)
MSH6	* 600678	Endometrial cancer, familial (AD), Lynch syndrome 5 (AD), Mismatch repair cancer syndrome 3 (AR)
MUTYH	* 604933	Adenomas, multiple colorectal (AR)
NBN	* 602667	Aplastic anemia, Leukemia, acute lymphoblastic, Nijmegen breakage syndrome (AR)
NF1	* 613113	Leukemia, juvenile myelomonocytic (AD), Neurofibromatosis-Noonan syndrome (AD), Neurofibromatosis, familial spinal (AD), Neurofibromatosis, type 1 (AD), Watson syndrome (AD)
NF2	* 607379	Schwannomatosis, vestibular (AD)
NTHL1	* 602656	Familial adenomatous polyposis 3 (AR)
PALB2	* 610355	Breast-ovarian cancer, familial, susceptibility 5 (AD), Pancreatic cancer, susceptibility 3 (AD), Fanconi anemia, complementation group N (AR)
PDGFRA	* 173490	Gastrointestinal stromal tumor/GIST-plus syndrome, somatic or familial, Hypereosinophilic syndrome,

		idiopathic, resistant to imatinib
PHOX2B	* 603851	Neuroblastoma, susceptibility 2, Central hypoventilation syndrome, congenital, 1, with or without Hirschsprung disease (AD), Neuroblastoma with Hirschsprung disease
PMS2	* 600259	Lynch syndrome 4, Mismatch repair cancer syndrome 4 (AR)
POLD1	* 174761	Colorectal cancer, susceptibility 10 (AD), Immunodeficiency 120 (AR), Mandibular hypoplasia, deafness, progeroid features, and lipodystrophy syndrome (AD)
POLE	* 174762	Colorectal cancer susceptibility 12 (AD), FILS syndrome (AR), IMAGE-I syndrome (AR)
POT1	* 606478	Cerebroretinal microangiopathy with calcifications and cysts 3 (AR), Pulmonary fibrosis and/or bone marrow failure syndrome, telomere-related, 8 (AD), Tumor predisposition syndrome 3 (AD)
PRKAR1A	* 188830	Acrodysostosis 1, with or without hormone resistance (AD), Carney complex, type 1 (AD), Myxoma, intracardiac (AD), Pigmented nodular adrenocortical disease, primary, 1 (AD)
PTCH1	* 601309	Basal cell nevus syndrome 1 (AD), Holoprosencephaly 7 (AD)
PTEN	* 601728	Glioma susceptibility 2 (AD), Meningioma (AD), Cowden syndrome 1(AD), Lhermitte-Duclos disease (AD), Macrocephaly/autism syndrome (AD)
RAD50	* 604040	Nijmegen breakage syndrome-like disorder (AR)
RAD51	* 179617	Breast cancer, susceptibility (AD), Fanconi anemia, complementation group R (AD), Mirror movements 2 (AD)
RAD51C	* 602774	Breast-ovarian cancer, familial, susceptibility 3, Fanconi anemia, complementation group O (AR)
RAD51D	* 602954	Breast-ovarian cancer, familial, susceptibility 4
RB1	* 614041	Retinoblastoma (AD), Retinoblastoma, trilateral (AD)
RECQL4	* 603780	Baller-Gerold syndrome (AR), RAPADILINO syndrome (AR), Rothmund-Thomson syndrome, type 2 (AR)
RET	* 164761	Hirschsprung disease, protection against (AD), Hirschsprung disease, susceptibility 1 (AD), Medullary thyroid carcinoma (AD), Multiple endocrine neoplasia IIA (AD), Multiple endocrine neoplasia IIB (AD), Pheochromocytoma (AD)
RNF43	* 612482	Sessile serrated polyposis cancer syndrome (AD)
RUNX1	* 151385	Leukemia, acute myeloid (AD), Platelet disorder, familial, with associated myeloid malignancy (AD)
SDHA	* 600857	Cardiomyopathy, dilated, 1GG (AR), Mitochondrial

		complex II deficiency, nuclear type 1 (AR), Neurodegeneration with ataxia and late-onset optic atrophy (AD), Pheochromocytoma/paraganglioma syndrome 5 (AD)
SDHAF2	* 613019	Pheochromocytoma/paraganglioma syndrome 2 (AD)
SDHB	* 185470	Gastrointestinal stromal tumor (AD), Mitochondrial complex II deficiency, nuclear type 4 (AR), Paraganglioma and gastric stromal sarcoma, Pheochromocytoma/paraganglioma syndrome 4 (AD)
SDHC	* 602413	Gastrointestinal stromal tumor (AD), Paraganglioma and gastric stromal sarcoma, Pheochromocytoma/paraganglioma syndrome 3 (AD)
SDHD	* 602690	Mitochondrial complex II deficiency, nuclear type 3 (AR), Paraganglioma and gastric stromal sarcoma, Pheochromocytoma/paraganglioma syndrome 1 (AD)
SLX4	* 613278	Fanconi anemia, complementation group P (AR)
SMAD4	* 600993	Juvenile polyposis/hereditary hemorrhagic telangiectasia syndrome (AD), Myhre syndrome (AD), Polyposis, juvenile intestinal (AD)
SMARCA4	* 603254	Otosclerosis 12 (AD), Rhabdoid tumor predisposition syndrome 2 (AD), Coffin-Siris syndrome 4 (AD)
SMARCB1	* 601607	Rhabdoid tumor predisposition syndrome 1 (AD), Schwannomatosis-1, susceptibility (AD), Coffin-Siris syndrome 3 (AD)
SMARCE1	* 603111	Meningioma, familial, susceptibility (AD), Coffin-Siris syndrome 5 (AD)
SPINK1	* 167790	Fibrocalculous pancreatic diabetes, susceptibility (AD, AR),Pancreatitis, hereditary (AD), Tropical calcific pancreatitis (AD, AR)
STK11	* 602216	Peutz-Jeghers syndrome (AD)
SUFU	* 607035	Medulloblastoma (AD, AR), Meningioma, familial, susceptibility (AD), Basal cell nevus syndrome 2, Joubert syndrome 32 (AR)
TERT	* 187270	Leukemia, acute myeloid (AD), Melanoma 9 (AD), Dyskeratosis congenita, 2 (AD, AR), Dyskeratosis congenita, (AD, AR), Pulmonary fibrosis and/or bone marrow failure syndrome, telomere-related, 1 (AD)
TMEM127	* 613403	Pheochromocytoma, susceptibility (AD)
TP53	* 191170	Adrenocortical carcinoma, pediatric (AD), Basal cell carcinoma 7 (AD), Choroid plexus papilloma (AD), Colorectal cancer, (AD), Glioma susceptibility 1 (AD), Osteosarcoma, Bone marrow failure syndrome 5 (AD), Li-Fraumeni syndrome (AD)

TSC1	* 605284	Tuberous sclerosis-1 (AD)
TSC2	* 191092	Tuberous sclerosis-2 (AD)
VHL	* 608537	Erythrocytosis, familial, 2 (AR), Pheochromocytoma (AD), von Hippel-Lindau syndrome (AD)
WT1	* 607102	Denys-Drash syndrome (AD), Frasier syndrome (AD), Meacham syndrome (AD), Nephrotic syndrome, type 4 (AD), Wilms tumor, type 1 (AD)
XRCC2	* 600375	Fanconi anemia, complementation group U (AR), Premature ovarian failure 17 (AR), Spermatogenic failure 50 (AR)

Clinically actionable genes are bolded. AD – autosomal dominant, AR – autosomal recessive, XLR – X-linked recesive
