# Supplementary material for: Hereditary Cancer Gene Panel Testing in a Croatian Cohort: The First Results from the University Hospital of Split
Source: Medicina (Kaunas). 2026 Sep 27;62(10):1870. doi: 10.3390/medicina62101870 (PMC13707095; doi:10.3390/medicina62101870)
Supplement: Supplementary file 1 [file medicina-62-01870-s001.zip › Supplementary Table S2.pdf]

Supplementary Table S2. Carriers of 2 Variants Reported as a Positive Finding

Patient	Gene	Variant	Variant Type	Phenotype	Age (years)
Patient 1	CDKN2A<br>FANCD2	c.250G>A, p.(Asp84Asn)<br>c.2487C>G p.(Tyr829Ter)	missense<br>nonsense	Melanoma	59
Patient 2	CDKN2A<br>FANCD2	c.250G>A, p.(Asp84Asn)<br>c.2487C>G, p.(Tyr829Ter)	missense<br>nonsense	Asymptomatic	38
Patient 3	BRCA1<br>ATM	c.181T>G, p.(Cys61Gly)<br>c.3802del, p.(Val1268Ter)	missense<br>frameshift	Asymptomatic	45
Patient 4	BRCA2<br>NTHL1	c.6641dup, p.(Tyr2215LeufsTer10)<br>c.244C>T, p.(Gln82Ter)	frameshift<br>nonsense	Breast cancer	54
Patient 5	MSH3<br>MUTYH	c.2319-1G>A<br>c.1353_1355del, p.(Glu452del)	splicing<br>inframe deletion	Ovarian cancer	62
Patient 6	BARD1<br>MUTYH	c.1224_1227del, p.(Met408IlefsTer6)<br>c.452A>G, p.(Tyr151Cys)	frameshift<br>missense	Breast cancer	58
Patient 7	TP53<br>CHEK2	c.(?_30)_(*220_?)del<br>c.470T>C p.(Ile157Thr)	large deletion<br>missense	Breast cancer, Chondrosarcoma	28
