# Supplementary material for: Hereditary Cancer Gene Panel Testing in a Croatian Cohort: The First Results from the University Hospital of Split
Source: Medicina (Kaunas). 2026 Sep 27;62(10):1870. doi: 10.3390/medicina62101870 (PMC13707095; doi:10.3390/medicina62101870)
Supplement: Supplementary file 1 [file medicina-62-01870-s001.zip › Supplementary Table S3.pdf]

Supplementary Table S3. List of All Variants Reported as a Positive Finding in Our Cohort

Gene	Variant	Variant Type	Number of carriers
APC	c.1652T>A, p.(Leu551Ter)	nonsense	1
ATM	c.9112C>T, p.(Gln3038Ter)	nonsense	1
ATM	c.3802del, p.(Val1268Ter)	frameshift	1
ATM	c.5932G>T, p.(Glu1978Ter)	nonsense	1
BARD1	c.1224_1227del, p.(Met408IlefsTer6)	frameshift	1
BRCA1	c.843_846del, p.(Ser282TyrfsTer15)	frameshift	2
BRCA1	c.181T>G, p.(Cys61Gly)	missense	2
BRCA1	c.1252G>T, p.(Glu418Ter)	nonsense	2
BRCA1	c.5266dup, p.(Gln1756ProfsTer74)	frameshift	6
BRCA1	c.4113del, p.(Cys1372ValfsTer21)	frameshift	2
BRCA2	c.9371A>T, p.(Asn3124Ile)	missense	3
BRCA2	c.6641dup, p.(Tyr2215LeufsTer10)	frameshift	5
BRCA2	c.5303_5304del, p.(Leu1768ArgfsTer5)	frameshift	1
BRCA2	c.7975A>G, p.(Arg2659Gly)	missense	1
BRCA2	c.1832C>G, p.(Ser611Ter)	nonsense	1
CDKN2A	c.71G>C, p.(Arg24Pro)	missense	1
CDKN2A	c.250G>A, p.(Asp84Asn)	missense	3
CHEK2	c.470T>C, p.(Ile157Thr)	missense	6
CHEK2	c.1232G>A, p.(Trp411Ter)	nonsense	2
CHEK2	c.(908+1_909-1)_(1095+1_1096-1)del	large deletion	1
DDX41	c.1141A>T, p.(Lys381Ter)	nonsense	1
EPCAM	c.(556-?_556+1)_(945+?_*?)del	large deletion	1
ERCC3	c.325C>T, p.(Arg109Ter)	nonsense	2
FANCC	c.487_490del, p.(Glu163IlefsTer30)	frameshift	1
FANCD2	c.2487C>G, p.(Tyr829Ter)	nonsense	2
FANCL	c.903+1_903+2del	splicing	1
FANCL	c.2T>C, p.(Met1?)	start loss	3
FANCM	c.2215_2216del, p.(Trp739AlafsTer11)	frameshift	1
HOXB13	c.251G>A, p.(Gly84Glu)	missense	1
LZTR1	c.1018C>T, p.(Arg340Ter)	nonsense	1
MLH1	c.(677+1_678-1)_(1667+1_1668-1)del, p.?	large deletion	1
MSH3	c.2319-1G>A	splicing	2
MSH6	c.3982C>T, p.(Gln1328Ter)	nonsense	1
MUTYH	c.452A>G, p.(Tyr151Cys)	missense	2
MUTYH	c.1353_1355del, p.(Glu452del)	inframe deletion	2
MUTYH	c.1103G>A, p.(Gly368Asp)	missense	1
NBN	c.657_661del, p.(Lys219AsnfsTer16)	frameshift	2
NBN	c.37+1G>A	splicing	1
NTHL1	c.244C>T, p.(Gln82Ter)	nonsense	4
PALB2	c.395del, p.(Val132AlafsTer45)	frameshift	3
PALB2	c.2470dup, p.(Cys824LeufsTer2)	frameshift	1
PALB2	c.3549C>G, p.(Tyr1183Ter)	nonsense	2
PALB2	c.172_175del, p.(Gln60ArgfsTer7)	frameshift	1

PMS2	c.1A>G, p.(Met1?)	start loss	1
POT1	c.1481_1484del, p.(Ile494LysfsTer15)	frameshift	1
RAD51C	c.724G>A, p.(Asp242Asn)	missense	1
TP53	c.475G>C, p.(Ala159Pro)	missense	1
TP53	c.(?_30)_(*220_?)del	large deletion	1
TSC1	c.1777del, p.(Arg593GlufsTer36)	frameshift	1
TSC2	c.3733del, p.(Arg1245GlyfsTer80)	frameshift	1
